# Multimodality diagnostic imaging and role of interventional radiology in pancreas transplantation

**DOI:** 10.1007/s00261-025-05003-w

**Published:** 2025-05-29

**Authors:** Hideyuki Torikai, Jordan Swensson, Eric Fromke, Klaus Hagspiel, John Angle, Rachita Khot

**Affiliations:** 1 University of Virginia Health System, Charlottesville, VA USA; 2 Indiana University, Bloomington, IN USA

**Keywords:** Pancreas transplant, CT, MRI, Ultrasound, Contrast-enhanced ultrasound, Interventional radiology

## Abstract

Pancreas transplantation remains a key therapeutic option for patients with type 1 diabetes mellitus. Despite advances in medical management, post-transplant complications continue to pose significant risks to graft survival, making early detection essential for optimizing patient outcomes. Given the complexity of pancreas transplantation, multimodality imaging plays a central role in evaluating graft function and identifying complications. Each modality provides pertinent information based on the complication suspected. This review outlines imaging strategies essential for assessing graft viability, distinguishing expected postoperative findings from pathologic conditions, and recognizing early signs of graft dysfunction. It also highlights the expanding role of interventional radiology, emphasizing minimally invasive techniques for effectively managing post-transplant complications and promoting better graft survival. By integrating current imaging protocols with interventional methodologies, this review offers radiologists and transplant clinicians a practical framework to optimize clinical decision-making, improve complication management, and enhance long-term graft outcomes.

**Supplementary Information:**

The online version contains supplementary material available at 10.1007/s00261-025-05003-w.

## Introduction

Pancreas transplantation is a definitive treatment for selected patients with insulin-dependent diabetes mellitus, particularly type 1 diabetes mellitus (T1DM), offering the potential for insulin independence and improved glycemic control [1]. The procedure is predominantly performed as a simultaneous pancreas and kidney transplant (SPK) in patients with diabetes-induced end-stage renal disease (ESRD), but can also be performed as a pancreas transplant alone (PTA) or a pancreas after kidney (PAK) transplant [2]. Advances in surgical techniques, immunosuppressive therapy, and post-transplant monitoring have significantly improved graft survival rates and patient outcomes. Despite these advances, complications associated with pancreas transplants remain significant contributors to graft loss [1, 3, 4]. Given the complexity of pancreas transplantation and its associated complications, comprehensive multimodality imaging assessment and interventional radiology procedures play an important role in the early detection and management of these complications, ensuring optimal graft function and long-term success.

## Epidemiology and indication

Over 2000 pancreas transplantations are performed annually worldwide [5]. The five-year graft survival rate for pancreas transplants has improved significantly with the development of surgical techniques and immunosuppression regimens, with graft survival rates of approximately 70–80% [1, 5, 6]. SPK is the most common form of pancreas transplantation. In 2023, approximately 89% of SPK pancreas transplantation surgeries were performed in the USA [2]. SPK provides dual benefits of improved glycemic control and renal replacement therapy, leading to better metabolic stability and a lower graft failure rate. PAK is the least common type of pancreas transplantation and is indicated for patients with T1DM and functioning kidney transplant but poorly controlled diabetes. PAK offers the benefit of restoring normoglycemia and a similar survival rate to that of SPK. PTA is considered for select patients with T1DM, cystic fibrosis, or status post total pancreatectomy without ESRD. This procedure remains less common due to the relatively high rate of graft loss within 1-year posttransplant [2].

## Anatomy and surgical techniques

**Fig. 1:**
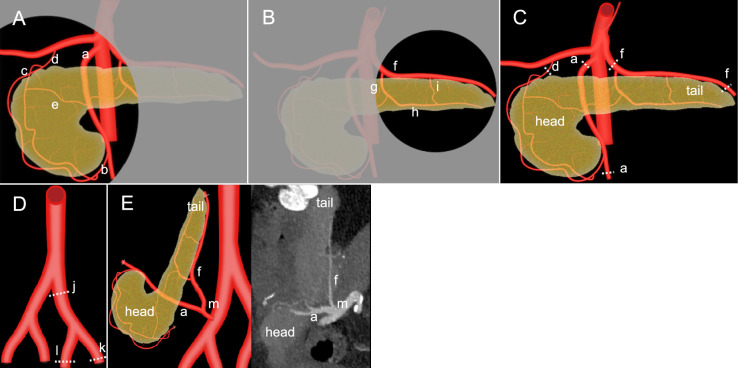
Arterial perfusion and anastomosis of the pancreas transplant. The illustration **A** demonstrates that the ventral pancreas is supplied by the superior pancreaticoduodenal artery (c), which arises from the gastroduodenal artery (d), and the inferior pancreaticoduodenal artery (b), which arises from the superior mesenteric artery (a, SMA). The illustration **B** demonstrates that the dorsal pancreas is supplied by the Splenic artery (f, SpA) and its branches, including the dorsal pancreatic artery (g), transverse pancreatic artery (h), and great pancreatic artery (i). A small anastomotic artery (e) connects the ventral and dorsal arterial systems (**A**). Resection of donor pancreas graft artery (**C**) and donor iliac Y-graft (**D**) for transplantation is shown with the dashed lines, where (j) is the common iliac artery, (k) is the external iliac artery, and (l) is the internal iliac artery. The illustration and the corresponding computed tomography angiography **E** demonstrate that the divided proximal SMA and SpA are anastomosed with the recipient’s common iliac artery (m) using the donor iliac Y-graft

Pancreas transplantation involves three types of anastomoses—arterial, venous, and exocrine. Complications are often observed at the anastomotic site. Therefore, knowing the anatomy of the pancreas helps recognize early signs of complications and is vital for timely intervention. Embryologically, the pancreas originates from separate ventral and dorsal buds, which fuse during development. Hence, the ventral pancreas (pancreatic head and uncinate process) and dorsal pancreas (pancreatic body and tail) receive separate arterial perfusion. The ventral pancreas and the duodenum are supplied by the superior pancreaticoduodenal artery (SPDA) arising from the gastroduodenal artery (GDA) and the inferior pancreaticoduodenal artery (IPDA) arising from the superior mesenteric artery (SMA). The SPDA and IPDA constitute an arterial network surrounding the ventral pancreas called the *pancreaticoduodenal arcade* (Fig. 1A). The dorsal pancreas receives blood supply predominantly from the branches of splenic artery (SpA), including the dorsal pancreatic artery (DPA), great pancreatic artery (pancreatic magna), transverse pancreatic artery (TPA), and pancreatic tail artery. The DPA typically originates from the proximal SpA and less commonly from the SMA or celiac trunk, perfusing the pancreatic body (Fig. 1B). [7].

During pancreas graft procurement surgery, the SMA is divided at its origin from the aorta, and the proximal SpA is divided to maintain a sufficient length for the subsequent arterial reconstruction. Additionally, the GDA and the portion of the SMA distal to the IPDA are ligated and divided. The distal SpA, located beyond the pancreatic tail branch near the splenic hilum, is ligated, allowing spleen separation (Fig. 1C). After the procurement of the whole pancreas graft, the donor’s common iliac artery (CIA) is harvested with its bifurcation into external and internal iliac arteries (EIA and IIA, respectively) to create an arterial Y-graft during back-table surgery (Fig. 1D). Subsequently, the EIA and IIA segments of the Y-graft are anastomosed to the SMA and SpA of the graft, respectively. Pancreas graft implantation commonly occurs in the recipient’s right pelvic fossa, where the donor CIA segment of the reconstructed Y-graft is typically anastomosed end-to-side with the recipient’s right CIA or EIA (Fig. 1E) [8–10]. Adequate perfusion through the SMA is critical for viability of the ventral pancreas and duodenal components, whereas perfusion via the SpA ensures dorsal pancreas graft function. Although collateral circulation exists via anastomoses between branches, such as the DPA and pancreaticoduodenal arcade, maintaining patency of both arterial systems is paramount for optimal graft survival and function.

**Fig. 2:**
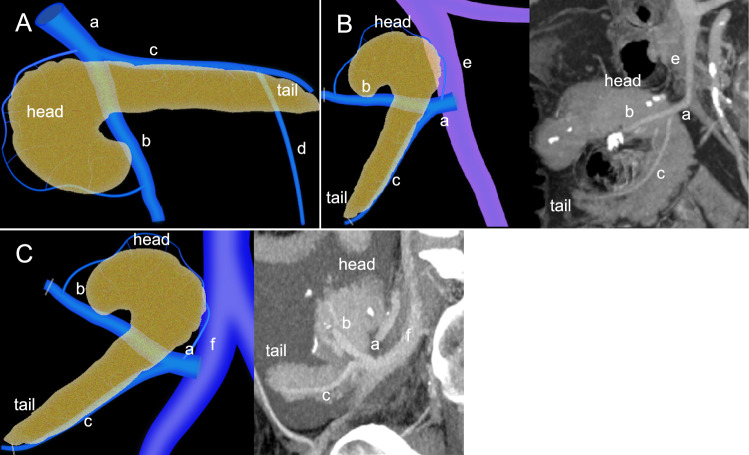
Normal venous anatomy and venous anastomosis. The pancreas has two separate venous drainage pathways. The illustration **A** demonstrates that the ventral pancreas drains into the superior mesenteric vein (b), and the dorsal pancreas drains into the splenic vein (c). The illustration and corresponding computed tomography (CT) images **B** demonstrate that the donor portal vein (a) is anastomosed with the recipient’s portal venous system which is the superior mesenteric vein (e). In a different case, the illustration and corresponding CT image **C** demonstrate that the recipient’s systemic venous system, where the donor portal vein (a) is anastomosed with the recipient’s iliac vein (f). (d) represents the inferior mesenteric artery in illustration (**A**)

There are two separate pancreatic venous drainage systems. The ventral pancreas drains into the superior mesenteric vein (SMV) and the dorsal pancreas into the splenic vein (SpV). The SMV and SpV joins to form the portal vein (PV) (Fig. 2A). Maintaining patency of these veins is critical for graft function. During the graft procurement surgery, the inferior mesenteric vein is ligated and divided. The distal SMV and SpV are also ligated and divided. The main PV is resected above the duodenum to leave a sufficient length for subsequent venous anastomosis [8, 9]. Two types of venous anastomosis are commonly performed, PV drainage (Fig. 2B) and systemic venous drainage (Fig. 2C). Systemic venous drainage, typically into the iliac vein or inferior vena cava, is technically more accessible and thus preferred over PV drainage [5]. However, PV drainage is considered more physiologic, as it routes pancreatic endocrine secretions through the liver before entering systemic circulation. Some authors suggest this may mitigate adverse effects from hyperinsulinemia associated with systemic drainage [10, 11]. Additionally, previous studies have suggested a possible immunological advantage of PV drainage [10, 12]. Nonetheless, there are no significant differences in glycemic control, metabolic parameters, and graft survival between the two approaches [11].

**Fig. 3:**
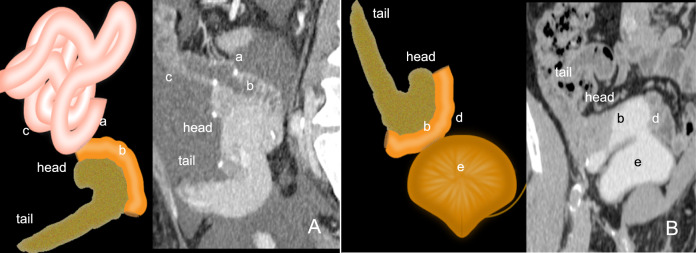
Exocrine drainage of the pancreas transplant. The illustration and corresponding computed tomography images (**A**) demonstrate enteric drainage via a duodenoenteric anastomosis (a). The (b) corresponds to the duodenal graft, and (c) corresponds to the small bowel. The illustration and corresponding computed tomography images (**B**) demonstrate bladder drainage via a duodenovesical anastomosis (d). The (e) corresponds to the bladder

For exocrine drainage of the pancreas graft, the donor duodenum is retrieved and transplanted en bloc with the pancreas. The IPDA typically supplies the duodenum; hence, careful retrieval is required to avoid injury to this artery, which could result in duodenal graft ischemia. There are two types of exocrine anastomosis, with similar graft and overall survival. Enteric drainage with duodenoenteric anastomosis (Fig. 3A) and bladder drainage with duodenovesical anastomosis (Fig. 3B). Enteric drainage accounts for over 90% of cases and is commonly constructed as a duodenojejunostomy. It provides more physiologic exocrine kinetics and is associated with a lower risk of metabolic complications [5]. The advantage of bladder drainage is the ability to detect pancreas graft rejection through urine analysis for pancreatic enzymes. Both types of exocrine drainage carry a risk for infection. Additionally, bladder drainage is associated with complications such as metabolic acidosis, reflux pancreatitis, and recurrent urinary infections. These complications lead to enteric conversion in up to 50% of cases [13].

## Imaging techniques and normal findings

### Ultrasonography

**Fig. 4:**
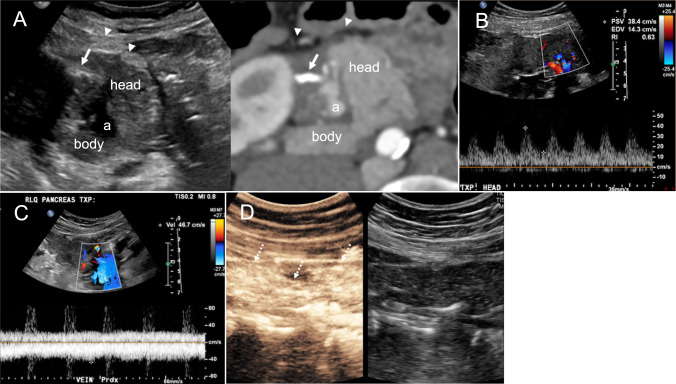
Ultrasound findings of a normal pancreas graft. Grayscale ultrasound with the corresponding computed tomography image for comparison **A** demonstrates a homogeneous hypoechoic pancreas graft head and tail surrounded by echogenic fatty tissue (arrowhead). The superior mesenteric vein (a) is seen as an anechoic structure near the pancreatic head. The echogenic line (arrow) corresponds to a surgical staple. Spectral Doppler of the artery **B** shows normal arterial waveforms with rapid systolic upstroke and continuous diastolic flow, and normal monophasic venous waveforms **C** in portal venous drainage. On contrast-enhanced ultrasound (**D**), the pancreas graft shows homogeneous enhancement within 10–20 s after contrast injection (dashed arrow)

Ultrasonography (US) is widely performed to evaluate pancreas grafts in perioperative, postoperative, outpatient, and emergency settings. However, it is technically challenging to visualize the entire graft and understand its orientation due to overlying bowel gas. On grayscale US, a normal pancreas graft appears homogeneous and hypoechoic, surrounded by hyperechoic fatty tissue and bowel gas (Fig. 4A). Color and spectral Doppler US are essential for vascular assessment. The SMA and SMV are located near the pancreatic head and appear as short anechoic tubular structures ending at the staple line. A staple line is seen as a linear echogenic band. The SpA and SpV courses along the pancreatic body and tail. On spectral Doppler, normal arterial waveform demonstrates a brisk systolic upstroke and continuous diastolic flow (Fig. 4B). Systolic velocities at the arterial anastomosis can reach up to 400 cm/s immediately post-transplant due to edema or arterial kinking. Over time, velocities typically normalize as edema resolves; however, persistently elevated velocities may suggest persistent kinking or stenosis [14, 15]. The resistive indices range from 0.5 to 0.7 [16], although there is no reliable measurement to suspect acute pancreas graft rejection due to the lack of a capsule [17]. Venous waveform varies by drainage type. PV drainage typically shows a monophasic waveform, while systemic venous drainage demonstrates mild cardiac phasicity [16] (Fig. 4C). The allograft venous velocities range from 10 to 60 cm/s [14].

Contrast-enhanced US (CEUS) enables evaluation of graft perfusion using a purely intravascular contrast agent. Unlike spectral Doppler, which assesses focal vascular segments, CEUS allows a holistic evaluation of entire parenchymal enhancement. The contrast agents can safely be used in patients with poor renal function or end-stage renal disease (ESRD) due to their lack of renal excretion. As a part of the US examination, CEUS can be performed at any time, immediately after transplant at the bedside or during outpatient follow-up. After a routine ultrasound, 2.4 mL or less of contrast is injected intravenously, followed by a 10 mL saline flush. Continuous imaging is performed for the first two minutes to evaluate arterial enhancement and detect subsequent washout. If rapid venous washout is not observed, intermittent imaging continues for 3–5 min to assess for contrast retention. The initial injection typically assesses the pancreatic head and body regions. A second intravenous injection is administered as necessary to confirm initial findings, evaluate pancreatic tail perfusion, or further investigate an abnormal area previously identified. Normally, the pancreas graft parenchyma exhibits uniform arterial enhancement, typically occurring within 10–20 s of contrast administration. (Fig. 4D) [18]. 

### Computed tomography

**Fig. 5:**
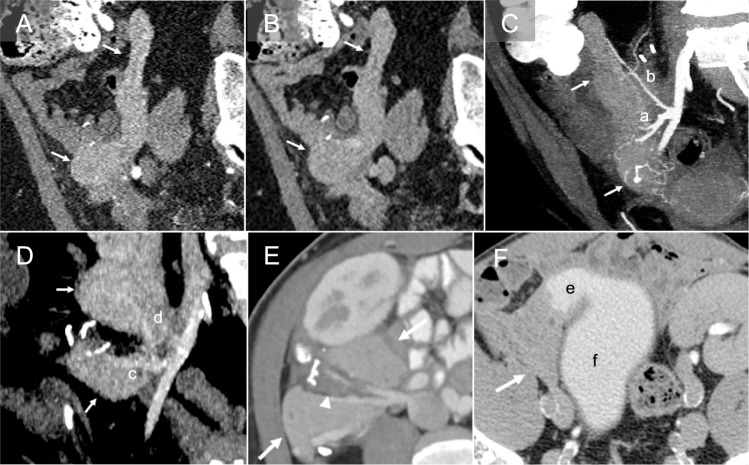
Normal findings on computed tomography (CT). A pancreas graft (arrow) demonstrates homogeneous enhancement during the arterial (**A**) and delayed (**B**) phases. CT angiography demonstrates the branching and course of the pancreas graft arteries (**C**) and veins (**D**): superior mesenteric artery (a), splenic artery (b), superior mesenteric vein (c), and splenic vein(d). Mild fat stranding in the graft mesentery is likely due to lymphatic edema and is a normal finding in the immediate postoperative setting (arrowhead, **E**). CT urography (**F**) shows contrast excretion into the mildly distended duodenal graft (e) and the recipient’s bladder (f) and delineates the anatomy

Computed tomography (CT) allows for obtaining sub-millimeter volume data, high-resolution multiplanar image reconstruction, and reconstructing three-dimensional (3D) images. This enables evaluation of the pancreas graft parenchyma, vascular structures, and complications in different planes. Multiphase CT angiography (CTA) with late arterial and venous phases is key to visualize vascular structures. Positive oral contrast can be used to evaluate bowel complications, such as duodenoenteric leak [3]; however, careful application is warranted due to possible obscuration of gastrointestinal bleeding [16]. Although the value of CTA for the pancreas graft is promising, intravenous iodine contrast administration is preferably avoided in patients with ESRD. Normal pancreas graft demonstrates homogeneous arterial (Fig. 5A) and delayed (Fig. 5B) enhancement with minimal visualization of the non-dilated main pancreatic duct. The uniform enhancement and course of the arteries **(**Fig. 5C**)** and veins **(**Fig. 5D) are well delineated on the arterial and delayed phases. Focal fat stranding of the residual graft mesentery is normal, which is thought to occur because of ligation of the donor’s lymphatics. Therefore, cautious assessment of parenchymal and peri-parenchymal changes is crucial to avoid misdiagnosing the findings as acute graft pancreatitis (Fig. 5E) [19]. The duodenovesical anastomosis and associated complications can be evaluated with CT cystography. CT cystography is performed by intravesical administration of approximately 400–500 mL of diluted iodine contrast via a Foley catheter to obtain sufficient bladder distention in our institute [20, 21]. CT urography (CTU) can be an alternative method; however, evaluation could be suboptimal due to insufficient bladder distention (Fig. 5F).

### Magnetic resonance imaging

**Fig. 6:**
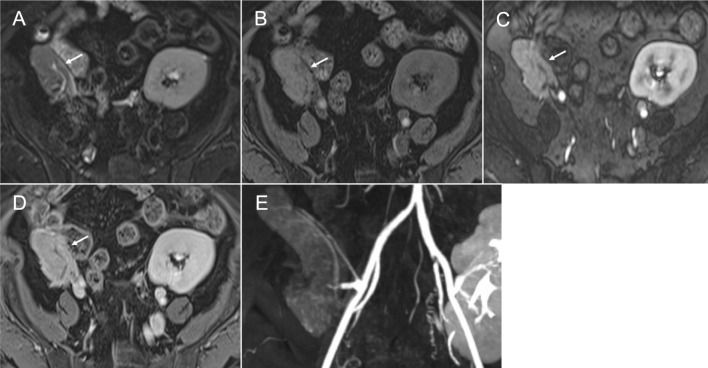
Normal findings on magnetic resonance imaging. A normal pancreas graft (arrow) demonstrates mildly high signal intensity on fat-suppressed (FS) T2-weighted imaging (**A**) and high signal intensity on FS T1-weighted imaging (**B**). Contrast-enhanced sequences show homogeneous enhancement on the arterial phase (**C**) and delay phase (**D**). Magnetic resonance angiography shows the course and smooth vascular contours of the transplant vasculature (**E**)

Magnetic resonance imaging (MRI) provides superior contrast resolution compared to CT and allows precise assessment of parenchymal changes. Since the risk of nephrogenic systemic fibrosis is now considered minimal with macrocyclic gadolinium-based contrast agents, they can be safely administered in patients with ESRD or those on dialysis who should avoid intravenous iodine contrast administration [22]. A normal pancreas graft demonstrates mildly high signal intensity on T2-weighted imaging (T2WI) (Fig. 6A) and intrinsically high signal intensity on fat-suppressed (FS) T1-weighted imaging (T1WI) (Fig. 6B) due to its proteinaceous content [3]. Post-contrast FS-T1WI demonstrates homogeneous enhancement on the arterial (Fig. 6C) and delayed phases (Fig. 6D). Some graft areas can be obscured by metallic artifacts from surgical staples. Magnetic resonance angiography (MRA) is also useful for evaluating vascular patency and complications (Fig. 6E) [23, 24]. The protocol at the institution of the lead author includes the following sequences: (1) Localizer, (2) Steady-state coherent sequence three planes, (3) Turbo spin echo (TSE) FS-T2WI axial, (4) Breath-holding 3D T1 DIXON Radiofrequency-spoiled gradient echo (Volumetric Interpolated Breath-hold Examination: VIBE) pre-contrast coronal, (5) Time-resolved MRA coronal, using timing bolus, i. 10 mL gadoterate meglumine injection, ii. Subsequent 20 mL saline flush, iii. Injection rate of 2 mL/s, (6) Breath-holding 3D MRA pre-contrast injection coronal, (7) Breath-holding 3D MRA post-contrast coronal with the same injection protocol, (8) 3D T1 DIXON VIBE post-contrast axial, (9) 3D T1 DIXON VIBE post-contrast coronal.

### Angiography

Conventional angiography remains a critical tool for managing vascular complications in pancreas transplantation, particularly when minimally invasive endovascular interventions are required. Vascular anastomosis in the pancreas graft is typically accessed via the unilateral common femoral artery or vein. The usefulness of 3D rotational angiography allows for precise spatial visualization of the vascular anatomy of the pancreas graft [25] (Supplementary Movie 1), and thus enhances preprocedural planning, reduces intervention times, and improves outcomes in thrombectomy or stent placement. Normal angiography of the pancreas graft demonstrates the smooth and regular Y-graft, intra- and extra-parenchymal arteries with homogeneous parenchymal enhancement during the arterial phase, followed by prompt opacification of the graft veins (Supplementary Movie 2).

## Complications

Outcomes following pancreas transplantation have continued to improve, with a decrease in mortality and increased graft survival rates. However, posttransplant complications and subsequent graft loss remain significant clinical challenge [2, 5]. These complications can be classified based on the structure involved, i.e., arterial, venous, perigraft, and graft parenchyma, as well as by timing, with early complications occurring within 3 months after transplantation and late complications thereafter [26]. Though these complications have been managed traditionally with surgical intervention, there has been a growing role for minimally invasive interventional radiology procedures in recent years. Therefore, appropriate imaging is essential for early diagnosis, preprocedural planning, and successful graft rescue.

### Arterial stenosis

**Fig. 7:**
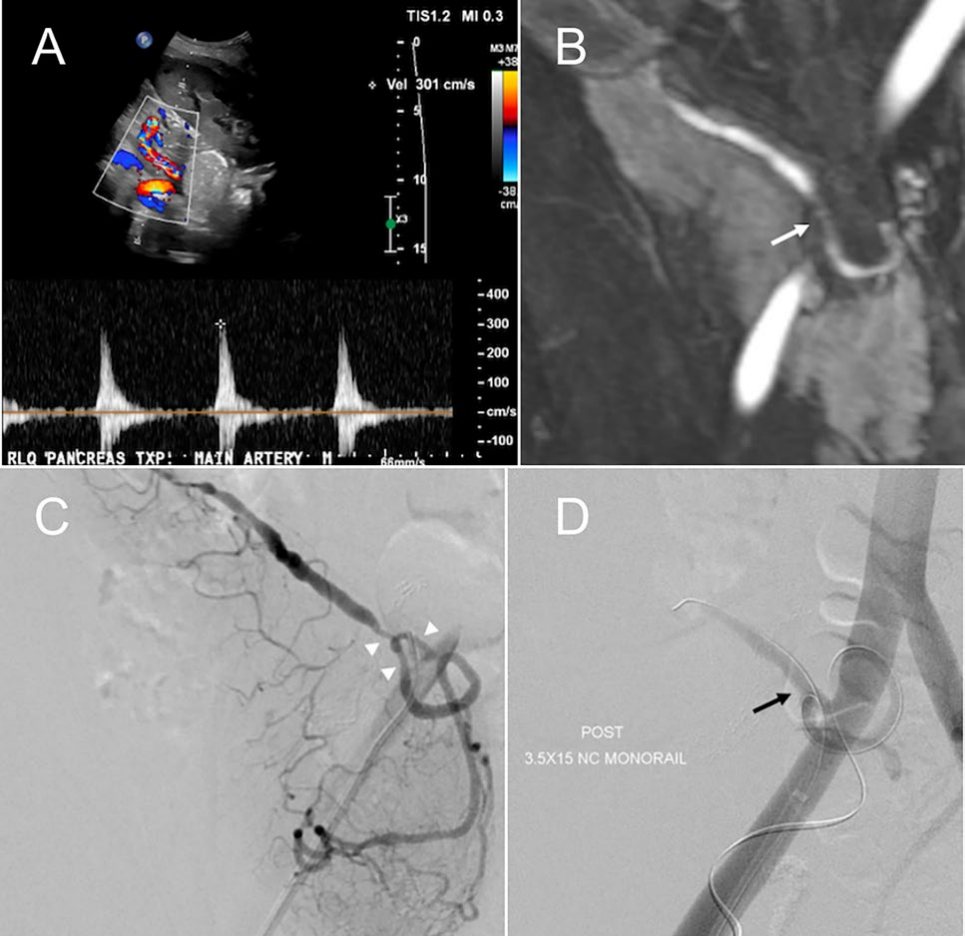
Arterial stenosis. A 51-year-old male status post simultaneous pancreas and kidney transplant for type 2 diabetes mellitus and diabetic nephropathy. Spectral Doppler ultrasound (**A**) performed at 3 months shows persistently elevated peak systolic velocity of 301 cm/s with turbulent flow at the anastomosis. Magnetic resonance angiography confirms the finding of stenosis at the anastomotic site (white arrow, **B**). Conventional angiography (**C**) also confirms the stenosis at the anastomosis (arrowheads). Post-balloon angioplasty angiography (**D**) shows improvement of the stenosis (black arrow)

Arterial stenosis is uncommon, with an incidence of approximately 2.5%, and is typically classified as an early post-transplant complication [27, 28]. It most often develops at the anastomotic site and can cause graft dysfunction, arterial thrombosis, ischemia, or necrosis. Contributing risk factors include surgical technique, graft kinking or twisting, acute rejection, ischemia at the anastomosis, and pre-existing atherosclerosis in the recipient’s iliac artery [23, 29, 30]. On Doppler US, clinically significant stenosis is suggested by turbulent flow and a peak systolic velocity exceeding 200–300 cm/s at the anastomosis (Fig. 7A). This elevated velocity persists on follow-up US as opposed to improvement in velocity seen over time with narrowing induced by postoperative edema. Additional findings in significant stenosis include a tardus parvus waveform, decreased resistive indices less than 0.5, and decreased peak systolic velocities in the graft arteries distal to the stenosis and within graft parenchyma [30, 31]. CEUS may show weak, non-uniform graft enhancement [18]. CTA or MRA and subsequent angiography can confirm the diagnosis and provide detailed anatomical information essential for planning endovascular intervention (Fig. 7B, C) [23, 24, 27, 31]. Percutaneous transarterial balloon angioplasty has demonstrated a 100% technical success rate and a graft survival rate of approximately 60% for hemodynamically significant stenosis (Fig. 7D) [32]. In cases involving Y-graft bifurcation stenosis, the double wire technique is useful for securing access to both true lumens [33]. In case of recipient iliac artery stenosis, stent placement may be required to restore adequate blood flow [34].

### Arterial thrombosis

**Fig. 8:**
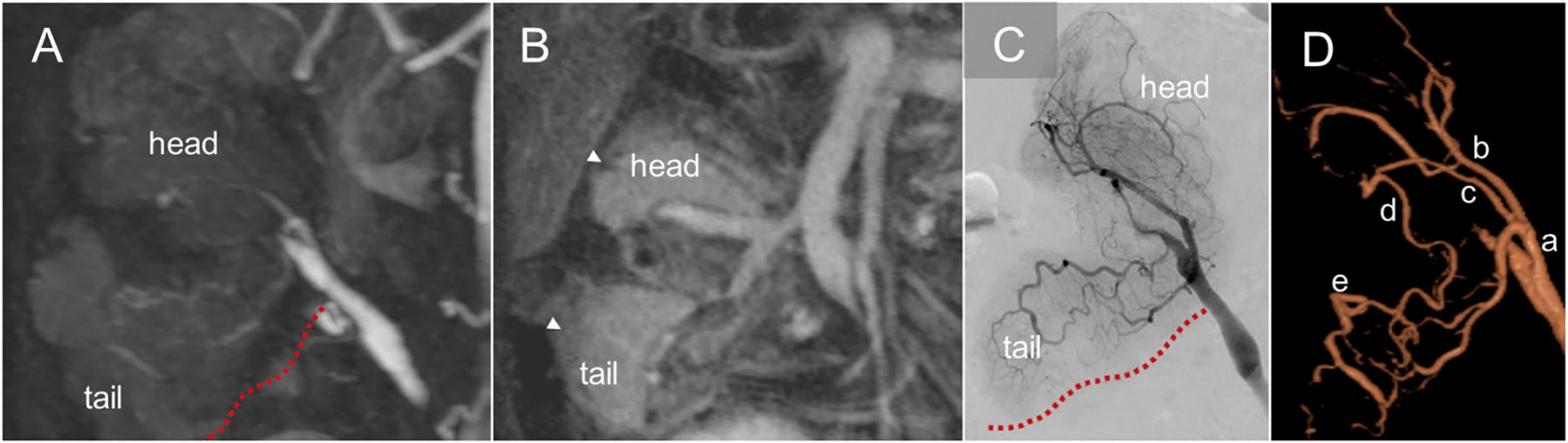
Arterial thrombosis and arterial arcade of the pancreas graft. A 45-year-old male with a history of type 1 diabetes mellitus and diabetic nephropathy status post pancreas after kidney transplantation. Magnetic resonance angiography (MRA) on postoperative day 38 during the arterial phase (**A**) demonstrates occlusion of the splenic artery (dotted red line). MRA during the venous phase (**B**) demonstrates normal parenchymal enhancement of the pancreatic tail (arrowhead) due to the intrapancreatic arterial network which avoids complete graft infarction. Conventional angiography (**C**) and 3D angiography (**D**) shows opacification of the intrapancreatic arterial network via the dorsal pancreatic artery (c), anastomotic artery (d), and transverse pancreatic artery (e). Notably, in this case, the dorsal pancreatic artery (c) arises from the superior mesenteric artery (a). (b) represents the inferior pancreaticoduodenal artery

Acute thrombotic complications of the graft vessel occur in 2–10% of pancreas transplant recipients, with arterial thrombosis accounting for approximately one-third to one-quarter of all thrombotic events. [4, 28, 35]. Arterial thrombosis is classified as an early posttransplant complication with risk factors including long back-table preparation and cold ischemia time, graft pancreatitis, and graft vessel injury. Thrombus formation often begins at the vessel stump due to stagnant blood flow, potentially progressing to involve parenchymal branches and leading to ischemic graft pancreatitis and dysfunction [16, 36, 37]. Complete arterial thrombosis can result in graft ischemia and necrosis; however, in limited cases of single vessel occlusion, either the SMA or SpA, some collateral flow through intrapancreatic networks can maintain the segmental arterial supply of the pancreas graft (Fig. 8) [38]. On grayscale US, arterial thrombosis appears as echogenic material within the transplant artery. Color Doppler US demonstrates either absent or diminished blood flow depending on the degree of arterial occlusion caused by the thrombus. The pancreas graft itself may show heterogeneous enlargement if ischemic pancreatitis or necrosis develops [3]. Although grayscale and Doppler US are valuable for detecting arterial thrombosis, image acquisition of the complete arterial Y-graft and branch arteries can be challenging due to bowel gas and the deep pelvic location of the graft.

**Fig. 9:**
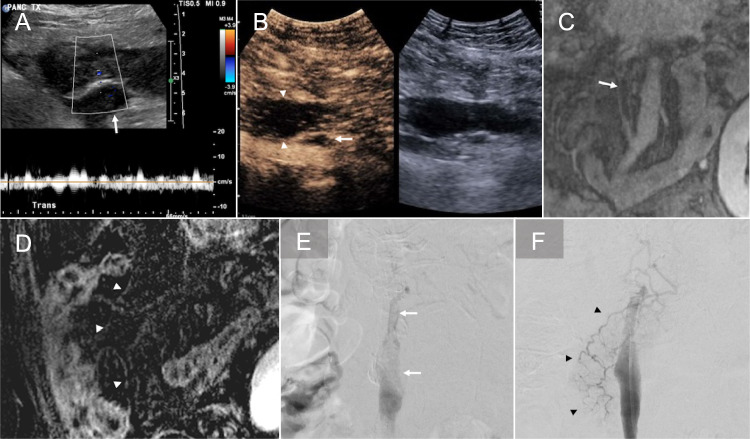
Arterial Thrombosis. **A** 44-year-old female with a history of type 1 diabetes mellitus status post pancreas transplant alone with elevated pancreatic enzymes. Color Doppler ultrasound (**A**) at the level stump of the Y graft demonstrates no arterial flow. Contrast-enhanced ultrasound (**B**) confirms the finding of thrombosis (arrow) as no contrast flows through the stump and no graft parenchymal enhancement (arrowhead). Magnetic resonance angiography (**C**) shows a filling defect in the stump of the Y arterial graft (arrow). Post-contrast magnetic resonance image (**D**) fat-suppressed T1-weighted imaging demonstrates loss of graft parenchymal enhancement (white arrowhead). Angiography (**E**) confirms an arterial thrombus as a filling defect (arrow) without parenchymal enhancement. Following percutaneous thrombectomy (**F**), contrast is seen in the stump of the Y graft and improved perfusion of the pancreas graft parenchyma (black arrowhead)

CEUS can directly evaluate graft parenchymal perfusion and is useful in differentiating very slow flow, where contrast is seen within the artery, and thrombosis, in which contrast is absent or minimal depending on the degree of occlusion [3, 18, 39]. Further evaluation using CTA or MRA provides a detailed assessment of thrombus extent, graft vascular anatomy, and parenchymal perfusion, facilitating targeted treatment planning [16, 23, 24]. Management of thrombosis with graft necrosis typically requires surgical intervention. In cases without parenchymal necrosis, anticoagulation therapy or minimally invasive endovascular approaches such as transarterial thrombolysis, mechanical thrombectomy, thrombus angioplasty, or catheter-directed thrombolysis with aspiration thrombectomy can be performed (Fig. 9) [32]. Due to the small caliber and tortuosity of transplant arteries, endovascular interventions have a relatively limited success rate [32].

### Arterial pseudoaneurysm

**Fig. 10:**
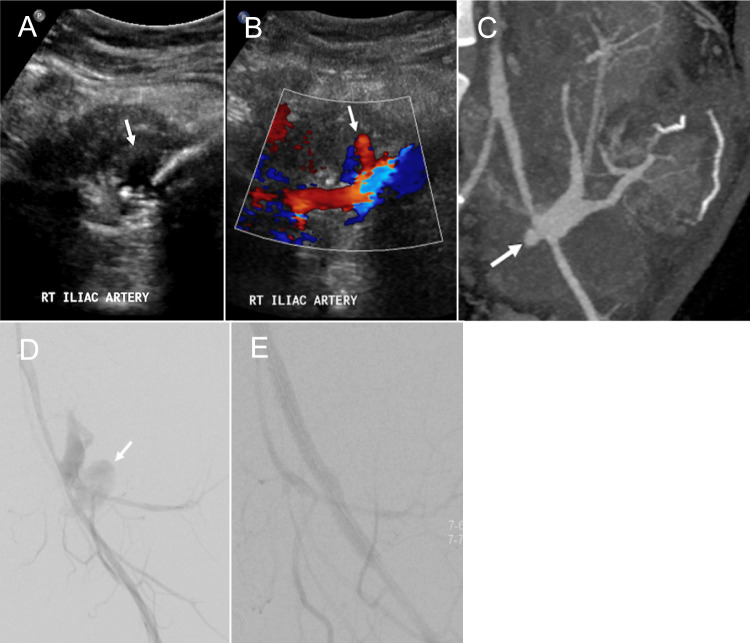
Arterial pseudoaneurysm (PSA). A 40-year-old male with a history of both type 1 and 2 diabetes mellitus and diabetic nephropathy underwent simultaneous pancreas-kidney transplantation, presenting with abdominal pain on postoperative day 40. Grayscale ultrasound (**A**) shows an anechoic cystic structure (arrow). Color Doppler ultrasound (**B**) reveals a characteristic Yin-Yang flow pattern (arrow) within the anechoic cystic structure, confirming the findings of PSA. CT angiography (**C**) demonstrates a saccular outpouching with enhancement (arrow). Angiography (**F**) confirms the outpouching filling with contrast due to the pseudoaneurysm (arrow) at the anastomotic site. Post-treatment angiography (**E**) demonstrates successful exclusion of the PSA following stent graft placement

Arterial pseudoaneurysm (PSA) and bleeding are among the most life-threatening complications of pancreas transplantation, with an incidence of approximately 2.4% [40]. These are usually caused by surgical trauma, pancreatitis, infection, or biopsy [27, 41]. PSA often occurs at the arterial anastomotic site and the biopsy site. Active hemorrhage may present as perigraft bleeding or intraluminal bleeding into the graft duodenum [4]. On grayscale ultrasound, a PSA is demonstrated as an anechoic cystic structure. Color Doppler US typically shows a turbulent “yin-yang” flow pattern, and spectral Doppler reveals a classic to-and-fro waveform at the PSA neck (Fig. 10A, B) [14, 16, 42]. CTA and MRA typically demonstrate a saccular outpouching with surrounding hematoma (Fig. 10C). These modalities are essential for precise localization of the PSA or bleeding site, which is helpful for transarterial embolization. As the PSA is at risk of impending rupture, immediate diagnosis and treatment are essential [3, 16, 30, 41, 42]. Endovascular treatment is often feasible in hemodynamically stable patients. Covered stent placement has been effective in excluding PSAs arising from the donor or graft iliac artery while preserving distal blood supply (Fig. 10D, E). Endovascular embolization with coils, plugs, or particles is an alternative treatment [28, 43]. Although technical success and hemostasis can often be achieved with endovascular treatment, complications and potential graft loss remain a concern [32, 43].

### Arteriovenous fistula

**Fig. 11:**
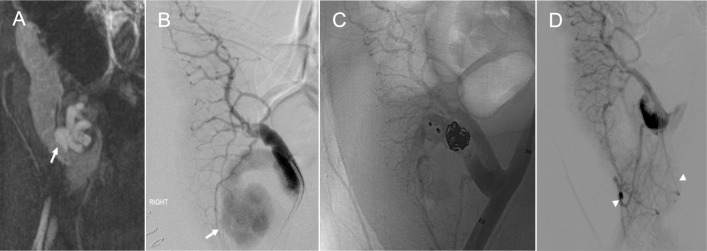
Arteriovenous fistula (AVF). A 30-year-old male with a history of type 2 diabetes mellitus is status post pancreas after kidney transplantation with delayed graft function on postoperative day 2. Magnetic resonance angiography (**A**) shows a tortuous, dilated AVF nidus (arrow) medial to the pancreas graft. Conventional angiography from the Y-graft artery (**B**) shows AVF nidus (arrow) and early draining vein involving the superior mesenteric artery and vein. Post-embolization angiography (**C-D**) shows complete occlusion of the AVF and improved enhancement of the pancreas graft (arrowhead)

Arteriovenous fistula (AVF) is a rare vascular complication following pancreas transplantation, with an estimated prevalence of approximately 1.4% [28]. AVF is defined as direct communication between an artery and a vein without an intervening capillary, resulting from simultaneous laceration of both arterial and venous walls and subsequent union of the lumens. This may occur during surgical procedures (e.g., blind ligation or stapling) or after a needle biopsy. AVFs are most commonly observed in the early post-transplant period but can develop at any time following transplantation or biopsy [30]. Clinically, they may lead to graft dysfunction or major bleeding. On spectral Doppler US, AVF demonstrates a high velocity, low resistance feeding artery, and arterialized biphasic pulsatile venous waveform. Persistent color Doppler aliasing at the maximum pulse repetition frequency setting is one of the most sensitive signs for detecting AVF [31]. Cross-sectional imaging with CTA or MRA shows early enhancement of the vein in continuity with the feeding artery on the arterial phase (Fig. 11A). A small AVF may resolve spontaneously, whereas a persistent or symptomatic AVF requires intervention. Successful transarterial embolization with or without transvenous procedure has been reported without major complications [32, 43]. Since some pancreatic branches can be identified proximal to the fistulous point, care must be taken to avoid embolizing these normal branches to preserve graft perfusion and minimize the risk of graft ischemia (Fig. 11B–D) [32].

### Venous thrombosis

**Fig. 12:**
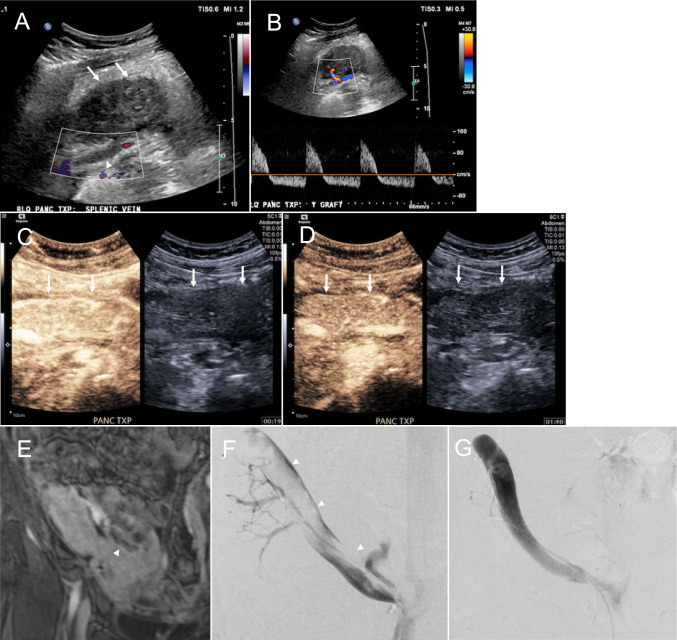
Venous thrombosis. A 44-year-old female with a history of type 1 diabetes mellitus underwent pancreas transplant alone with clinical presentation of increased blood in the urine on postoperative day 1. Color Doppler ultrasound (**A**) shows a heterogeneous appearing and enlarged pancreas graft (arrow) with no color flow within the graft vein due to thrombosis (arrowhead). Spectral Doppler ultrasound (**B**) of the intraparenchymal artery shows reversed diastolic arterial waveforms. Contrast-enhanced ultrasound demonstrates prompt parenchymal enhancement at 19 s after injection (arrow, **C**) and minimal washout at 100 s after injection (arrow, **D**). Magnetic resonance angiography (**E**) during venous phase confirms the presence of thrombus in the splenic vein (arrowhead). Pre-treatment venography (**F**) shows a filling defect corresponding to splenic vein thrombus (arrowhead). Post-treatment venography (**G**) shows resolution of the thrombus and restoration of venous blood flow

Venous thrombosis is the most common vascular complication and the second most common complication of graft dysfunction after rejection. It typically occurs within 4–6 weeks of transplantation and is considered an early postoperative complication [36]. Thrombosis is multifactorial in origin. Common causes include surgical challenges, venous stasis, anastomotic stricture, venous kinking, pancreatitis, and graft rejection [27, 30, 38]. While partial thrombosis may be asymptomatic, occlusive thrombosis may induce ischemic graft pancreatitis and infarction. Thrombus in the distal SpV or SMV can be incidentally detected, which can propagate towards the central venous system [16]. Grayscale US demonstrates a distended vein with echogenic thrombus. Decreased or absent blood flow is detected on color Doppler US, depending on the degree of occlusion. Reversal of the diastolic arterial waveform is observed in obstructive thrombosis on spectral Doppler US (Fig. 12A, B) [44]. The pancreas graft becomes enlarged and is heterogeneous due to congestion [45]. On CEUS, venous thrombosis presents as delayed washout of the contrast in the venous phase despite preserved arterial enhancement. Intraluminal non-enhancement of the graft vein further supports the diagnosis (Fig. 12C, D) [18]. CTA and MRA are helpful in assessing the extent of the thrombus. Thrombus is seen as an intraluminal filling defect, and often accompanied by edematous changes in the pancreas graft parenchyma (Fig. 12E) [23, 24, 27, 31, 37]. Partial thrombosis without parenchymal ischemia can be treated with anticoagulation [28, 36]. Whereas occlusive venous thrombosis without graft necrosis, endovascular interventions, such as mechanical thrombectomy and catheter-directed thrombolysis with aspiration, may be effective (Fig. 12F, G). Catheter-directed mechanical thrombectomy with or without chemical thrombolysis has shown a graft survival rate of up to 76% [46].

### Fluid collection

**Fig. 13:**
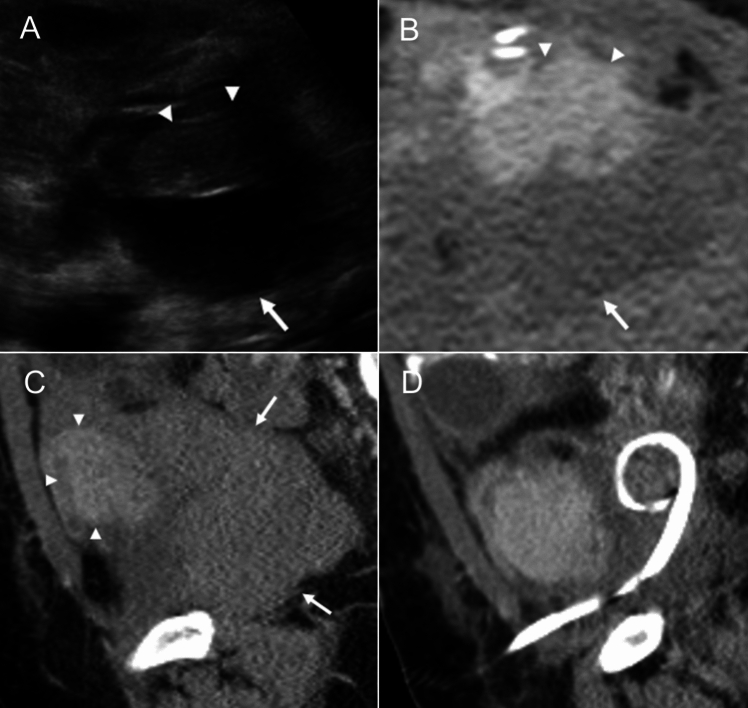
Peripancreatic fluid collection. A 31-year-old male with a history of type 1 diabetes mellitus and nephropathy underwent pancreas after kidney transplantation. Grayscale ultrasound (**A**) on postoperative day 14 demonstrates anechoic simple fluid collection (arrow) adjacent to the pancreas graft (arrowhead). Contrast-enhanced computed tomography (CT) (**B-C**) shows hypodense to isodense and non-enhancing fluid collection (arrow) adjacent to the pancreas graft (arrowhead). Pre-drainage CT (**C**) demonstrates the extent of the fluid collection, and post-drainage CT (**D**) shows a significant decrease in the size of the fluid collection following percutaneous drainage tube placement

Peripancreatic fluid collection occurs in approximately 11–16% of pancreas transplant recipients and is associated with reduced graft survival. These collections are seen in the early postoperative period but can occasionally present beyond 3 months after transplantation [47, 48]. Postsurgical seroma and lymphocele result from disruption of the lymphatics in the surgical bed [49]. Exocrine anastomotic leak may result in fluid collection, abscesses, or, less commonly, urinomas. Pseudocysts can develop in graft pancreatitis, while peripancreatic bleeding can cause hematoma or hemorrhagic fluid collections. On grayscale US, these collections typically appear as an anechoic cystic collection without an internal Doppler signal (Fig. 13A), whereas infectious or hemorrhagic collections may contain internal echogenic debris. On CT, simple fluid collections appear hypodense, while hemorrhagic collections demonstrate increased density (Fig. 13B) [3]. The presence of air bubbles can be seen in the infectious fluid collection, abscess, or enteric leakage. MRI helps further characterize fluid collections. Simple seromas or lymphoceles demonstrate high T2 signal intensity. Abscesses typically show peripheral rim enhancement on post-contrast images and restricted diffusion with corresponding low signal on the apparent diffusion coefficient map. Hemorrhagic collections vary in signal intensity depending on the age of the blood products [3, 41]. Percutaneous US or CT-guided drainage is a feasible treatment for these fluid collections (Fig. 13C, D). Around 80% of the cases can be effectively managed with image-guided percutaneous drainage alone, although surgical intervention may be required in refractory or complicated cases [50, 51].

### Exocrine anastomotic leak

**Fig. 14:**
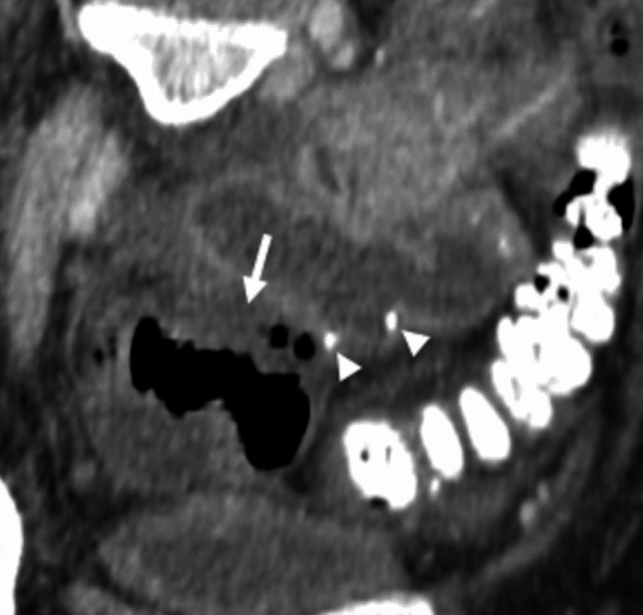
Doudenoenteric anastomotic leak. A 52-year-old male with a history of type 1 diabetes mellitus underwent pancreas after kidney transplantation, presented with abdominal pain and an elevated white blood cell count. Contrast-enhanced computed tomography demonstrates a nearly well-defined non-enhancing fluid collection (arrow) with foci of air adjacent to the doudenoenteric anastomosis (arrowhead) secondary to enteric leak. The fluid collection was treated with percutaneous drain placement

**Fig. 15:**
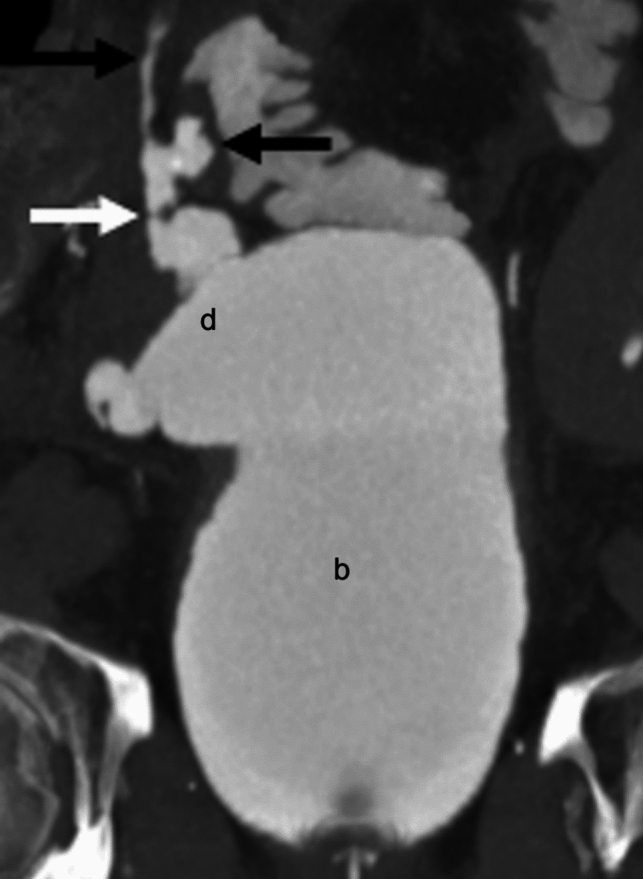
Duodenal stump urine leak following duodenovesical anastomosis. A 58-year-old male with a history of type 1 diabetes mellitus and diabetic nephropathy underwent simultaneous pancreas-kidney transplantation, presenting 10 years after transplantation with elevated pancreatic enzymes. A small fluid collection was seen on contrast-enhanced CT (not shown). CT cystography was performed, which demonstrated contrast outside the bladder (b) and duodenum graft (d) secondary to urine leak (black arrow) near the duodenal stump (white arrow)

Exocrine leak may occur at the duodenoenteric anastomosis, duodenovesical anastomosis or duodenal stump. The reported incidence is up to 20% of pancreas transplants [40, 52, 53] and is responsible for less than 1% of all causes of graft loss [2, 5, 11]. While the exocrine anastomotic leak is classified as an early postoperative complication, the leak from the duodenal stump may present later [48, 54]. The common symptoms of the leak are pain, fever, leukocytosis, peritonitis, and graft dysfunction. These symptoms are typically milder in patients with bladder drainage compared to those with enteric drainage [4, 35]. Although US and MRI are useful in detecting fluid collection associated with the exocrine anastomotic leak, CT is the gold standard for diagnosing the leak [55]. Fluid collection, extraluminal air, or extravasation of oral contrast is demonstrated adjacent to the doudenoenteric anastomosis or duodenal stump (Fig. 14) [56]. Secretin-stimulated MRI can be used to differentiate an anastomotic leak from pancreatitis-associated fluid collection [57]. Urine leak at the duodenovesical anastomosis or duodenal stump appears as fluid collections and extravasation of the intravesical contrast on CTU or CT cystography (Fig. 15) [20, 21]. Prompt surgical intervention or percutaneous drainage is required to avoid further complications for enteric leakage. Urine leak from duodenovesical anastomosis or duodenal stump without infection or peritonitis is often managed by bladder decompression and percutaneous drainage. Significant compromise of the duodenovesical anastomosis or the duodenal stump may require surgical repair [53].

### Graft pancreatitis

**Fig. 16:**
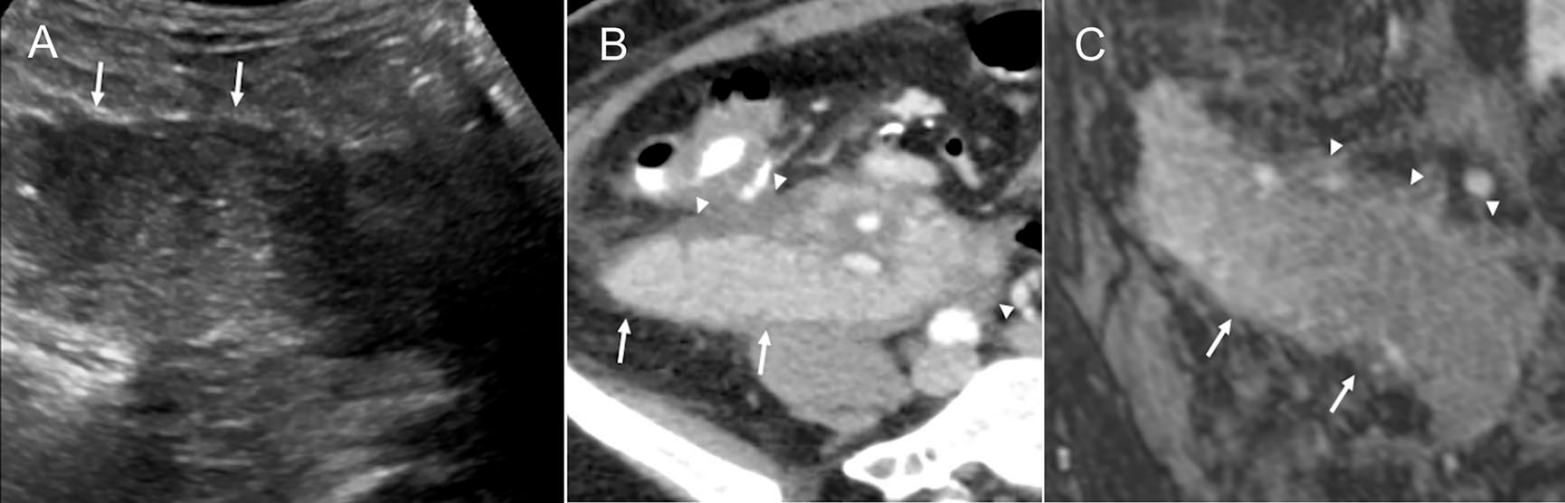
Acute graft pancreatitis. A 35-year-old female with a history of type 1 diabetes mellitus underwent pancreas transplantation alone and presented with persistently elevated blood glucose levels on postoperative day 12. Grayscale ultrasound (**A**) shows an enlarged pancreas graft with heterogeneous echotexture (arrow). Contrast-enhanced computed tomography (**B**) shows low-density acute inflammatory fluid (arrowhead) surrounding the pancreas graft (arrow), and magnetic resonance imaging (**C**) shows graft (arrow) enlargement with surrounding edematous changes (arrowhead). Rejection can have similar findings and is differentiated with biopsy

Graft pancreatitis is one of the most common complications following pancreas transplantation and is classified as acute or chronic [58, 59]. Acute graft pancreatitis is further categorized as physiological, early, and late. Physiological acute graft pancreatitis is a self-limiting process observed in all pancreas transplants. Early acute graft pancreatitis occurs within 3 months of transplantation. It is caused by infection, immunological reaction, or vascular thrombosis. It carries a higher risk of 1-year graft loss [4, 35]. Late acute pancreatitis occurring beyond 3 months may result from urinary reflux or outflow obstruction, parenchymal microvascular thrombosis, or infection. It has no known association with graft loss [58, 60]. Clinical symptoms of graft pancreatitis include pain, tenderness, systemic inflammatory response, or graft dysfunction. Necrotizing graft pancreatitis is the most severe form of pancreatitis, occurring in approximately 2–4% of cases. Acute graft pancreatitis or vascular complications can induce graft necrosis [15]. Imaging findings are similar to those of native pancreatitis. Grayscale US demonstrates an enlarged, heterogeneous, and hypoechoic graft (Fig. 16A), although these findings can also be seen in acute rejection and early thrombosis. Perigraft fluid collection can be detected as indirect findings [16, 27]. CT and MRI better delineates the enlarged pancreas graft with heterogeneous parenchymal enhancement and surrounding edema (Fig. 16B, C) [3, 27, 29, 31, 41]. These modalities demonstrate high sensitivity and specificity for detecting complications [29, 45]. Hypo- or non-enhancing graft parenchyma containing foci of air suggests necrotizing pancreatitis. This necrotic parenchyma may demonstrate absence of Doppler signal or lack of enhancement on CEUS [27, 42]. Acute graft pancreatitis can be managed conservatively, while necrotizing graft pancreatitis may require surgical management [4, 19]. Chronic graft pancreatitis is an overlapping entity of chronic rejection. It is associated with late graft loss in 4–10% of cases [58].

### Rejection

**Fig. 17:**
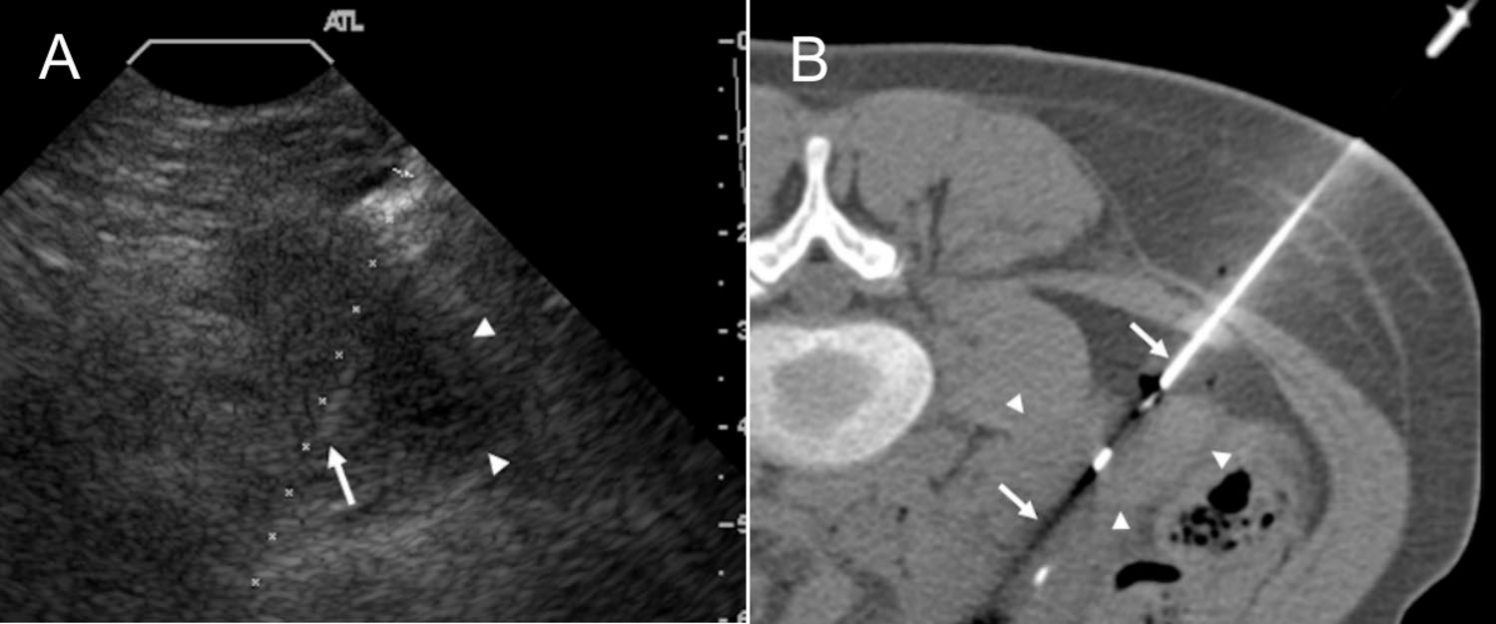
Image-guided biopsy for rejection. The Ultrasound (**A**) and computed tomography image (**B**) shows a longitudinal biopsy trajectory (arrow) along the long axis of the pancreas graft (arrowhead) to avoid vascular and ductal injury. Biopsy confirmed mild acute rejection

Pancreas allograft rejection is categorized as acute or chronic. The reported incidence of acute rejection is 5–25% [1, 5], and acute rejection is a significant risk factor for long-term graft loss [5]. On grayscale US, an enlarged pancreas graft with heterogeneous echotexture is observed. The primary role of the US is to exclude vascular thrombosis [3]. Unlike other organ transplants, elevated resistive indices have not been a useful indicator to confirm rejection [17]. Edematous parenchyma and surrounding structures with heterogeneous enhancement are demonstrated on CT and MRI, which are nonspecific findings and often seen in graft pancreatitis and graft thrombosis [3, 16, 27]. Chronic rejection can result from recurrent acute rejection episodes and subsequent graft fibrosis and acinar loss. It occurs 3–6 months after a pancreas transplant, with an estimated incidence of 4–10%. Chronic rejection is considered as the major cause of long-term graft failure [1, 3, 61]. Atrophic pancreas graft with increased echogenicity is observed on grayscale US. Decreased T1 signal intensity with delayed parenchymal enhancement reflecting fibrosis is demonstrated on MRI. Imaging findings of both acute and chronic rejection are nonspecific and overlap with other complications, which makes early diagnosis challenging [3, 41]. Given the lack of specific and reliable diagnostic findings, the image-guided percutaneous biopsy remains the gold standard for diagnosis. US guidance provides real-time monitoring of ductal and vascular structures for safe biopsy; however, overlying bowel gas limits the visibility of the graft. A CT-guided biopsy is feasible, although detection of ductal and vascular structures is limited. Previous reports have demonstrated a high successful diagnostic rate of 72–96% and a low major complication rate of 2.6–5.9% using up to an 18-gauge core biopsy needle under image guidance (Fig. 17) [62, 63]. The preferred biopsy trajectory is toward the tail longitudinally with respect to the long axis of the pancreas to avoid splenic vessels and the main pancreatic duct, given the large vessels and ducts in the head region [27, 64].

## Conclusion

Multimodality imaging combined with targeted interventional radiology plays an important role in the management of complications after pancreas transplantation. Familiarity with normal postoperative appearances and early recognition of abnormal findings are essential for timely interventions that preserve graft function and improve outcomes. Pancreas transplantation presents unique diagnostic and therapeutic challenges that require a comprehensive understanding of surgical anatomy, postoperative changes, and potential complications. As transplant techniques continue to evolve, integration of advanced imaging and interventional approaches will remain central to optimizing care in pancreas transplant recipients.

## Supplementary Information

Below is the link to the electronic supplementary material.

Supplementary file1 (MP4 4536 KB)

Supplementary file2 (MP4 2488 KB)

## Data Availability

No datasets were generated or analysed during the current study.

## Abbreviations

AVF: Arteriovenous fistula
CEUS: Contrast-enhanced ultrasonography
CIA: Common iliac artery
CT: Computed tomography
CTA: Computed tomography angiography
CTU: Computed tomography urography
DPA: Dorsal pancreatic artery
EIA: External iliac artery
ESRD: End-stage renal disease
FS: Fat-suppressed
IIA: Internal iliac artery
IPDA: Inferior pancreaticoduodenal artery
MRA: Magnetic resonance angiography
MRI: Magnetic resonance imaging
PAK: Pancreas transplant after kidney
PSA: Pseudoaneurysm
PTA: Pancreas transplant alone
PV: Portal vein
SMA: Superior mesenteric artery
SMV: Superior mesenteric vein
SpA: Splenic artery
SPDA: Superior pancreaticoduodenal artery
SPK: Simultaneous pancreas and kidney transplant
SpV: Splenic vein
TSE: Turbo spin echo
T1DM: Type 1 diabetes mellitus
T1WI: T1-weighted imaging
T2WI: T2-weighted imaging
US: Ultrasonography
VIBE: Volumetric interpolated breath-hold examination
3D: Three-dimensional
